# MCF-7 Drug Resistant Cell Lines Switch Their Lipid Metabolism to Triple Negative Breast Cancer Signature

**DOI:** 10.3390/cancers13235871

**Published:** 2021-11-23

**Authors:** Jose Adriá-Cebrián, Sandra Guaita-Esteruelas, Eric W.-F. Lam, Marta Rodríguez-Balada, Jordi Capellades, Josefa Girona, Ana Maria Jimenez-Santamaria, Oscar Yanes, Luís Masana, Josep Gumà

**Affiliations:** 1 Department of Oncology, Hospital Universitari de Sant Joan, E-43204 Reus, Spain; jose.adria@estudiants.urv.cat (J.A.-C.); mrodriguez@grupsagessa.com (M.R.-B.); anamaria.jimenez@salutsantjoan.cat (A.M.J.-S.); josep.guma@salutsantjoan.cat (J.G.); 2 Center for R&D&I in Nutrition and Health, Institut de Investigació Sanitaria Pere Virgili (IISPV), Avda. de la Universitat, 1—Second Floor, E-43204 Reus, Spain; jordi.capellades@estudiants.urv.cat (J.C.); josefa.girona@urv.cat (J.G.); oscar.yanes@urv.cat (O.Y.); luis.masana@urv.cat (L.M.); 3 Research Unit on Lipids and Atherosclerosis, Institut de Investigació Sanitaria Pere Virgili (IISPV), Universitat Rovira i Virgili, 43204 Reus, Spain; 4 Spanish Biomedical Research Centre in Diabetes and Associated Metabolic Disorders (CIBERDEM), Institute of Health Carlos III, 28029 Madrid, Spain; 5 State Key Laboratory of Oncology in South China, Collaborative Innovation Center of Cancer Medicine, Sun Yat-sen University Cancer Center, Guangzhou 510060, China; Eric.lam7314@gmail.com; 6 Department of Electronic Engineering, Universitat Rovira i Virgili, 43007 Tarragona, Spain

**Keywords:** breast, tumor microenvironment, adipocytes, FABP4, FABP5, CD36, lipids, mass spectrometry

## Abstract

**Simple Summary:**

Previously, we have demonstrated that lipid and lipoprotein profiles in breast cancer patients are altered compared to a control showing a new link between triglycerides enriched particles and breast cancer, hence suggesting an active role of lipids and their metabolism in this pathology. In this study, we have demonstrated the importance of tumor microenvironment crosstalk as well as the crucial role of lipid transfer between adipose tissue and cancerous cells. Moreover, we have demonstrated that each breast cancer subtype has their specific lipid signature. Interestingly, drug resistant luminal A cell lines switch this metabolic profile to the triple negative lipid signature. Knowledge of these signatures might help us to understand how these specific lipids are related with drug resistance and how it may clarify new treatments in these cancer patients.

**Abstract:**

Obesity and adipose tissue have been closely related to a poor cancer prognosis, especially in prostate and breast cancer patients. The ability of transferring lipids from the adipose tissue to the tumor cells is actively linked to tumor progression. However, different types of breast tumor seem to use these lipids in different ways and metabolize them in different pathways. In this study we have tracked by mass spectrometry how palmitic acid from the adipocytes is released to media being later incorporated in different breast cancer cell lines (MDA-MB-231, SKBR3, BT474, MCF-7 and its resistant MCF-7 EPI^R^ and MCF-7 TAX^R^). We have observed that different lines metabolize the palmitic acid in a different way and use their carbons in the synthesis of different new lipid families. Furthermore, we have observed that the lipid synthesis pattern varied according to the cell line. Surprisingly, the metabolic pattern of the resistant cells was more related to the TNBC cell line compared to their sensitive cell line MCF-7. These results allow us to determine a specific lipid pattern in different cell lines that later might be used in breast cancer diagnosis and to find a better treatment according to the cancer molecular type.

## 1. Introduction

Breast cancer is the most common cancer in women and the second one overall [1]. Despite the advance in diagnosis techniques and treatment, nearly 12% of breast cancer patients eventually develop metastasis, and die from the disease [2]. A number of risk factors contribute to the poor outcomes, and the lifestyle is one of the most important. Obesity is one of the main known risk factors involved in the development and progression of breast cancer, as well as other cancer types, including pancreatic, hepatic and gallbladder cancer [3]. Although for many years adipose tissue has been consider as an energy reservoir, recent studies have demonstrated the importance of fat in different biological and physiological processes, including the production of steroid hormones, cytokines and adipokines, the control of immune system, and the regulation of eating behavior [4]. Due to the rapid proliferation rates of tumor cells, they require a substantial amount of lipid molecules for the biosynthesis of membranes, organelles, and other cellular factors. Adipose tissue, as a principal source of lipids, has an important influence in cancer progression through the transfer of lipids [5]. In fact, a number of studies have demonstrated that adipose tissue modulates cancer progression via the releasement of different factors and by the transfer of diverse lipid molecules [6]. Most of these factors are released as a consequence of the close communication between adipose tissue and the cancer cells [7]. Several studies have already demonstrated that there is an interaction between tumor cells and their microenvironment, which includes adipocytes [8,9]. This crosstalk of information and materials is mediated by the release of molecules, such as cytokines, hormones, metabolites and growth factors, that act on the closely located cells, as well as the more distant cells, modifying their behavior by the activation or inhibition of different metabolic pathways and/or by altering their proteomes and transcriptomes [7,10].

The communication between tumor cells and the adipocytes is bidirectional, and it has been described that tumor cells are able to modify the transcriptomes and metabolism of adjacent cells for their own benefit. In fact, adipocytes located within the tumor microenvironment are also subjected to dedifferentiation and a reprogramming of their metabolic behavior, transforming into cancer associated adipocytes (CAAs) [11]. CAAs release metabolites, such as lactate, pyruvate, free fatty acids, and ketone bodies, to the extracellular matrix, fuelling the proliferation of adjacent tumor cells [12]. Tumor cells internalized these lipids into their metabolic pathways for oxidation and energy production, and for activation of different signaling pathways [9,13].

Moreover, lipids are a high heterogenous variety of molecules. Depending on their structure and composition, lipids can play a plethora of roles in cell biology, such as membrane biosynthesis, energy storage, and cell signal transmission [14,15].

According to the Lipid Maps Alliance, lipids can be divided into eight different groups: fatty acids (FAs), glycerolipids (GL), glycerophospholipids (GP), sphingolipids (SP), sterol lipids (ST), prenol lipids (PR), saccharolipids (SL), and polyketides (PK) [16]. It is well established that tumor metabolism is subject to a number of modifications, and a switch to a lipidic program enhances tumor malignant transformation and progression [17].

These tumor lipid metabolic modifications also provide the possibilities for novel diagnostic strategies in cancer. As lipids can be found in urine, blood and other different accessible tissues, a lipid molecular screening by lipidomic technology can aide cancer diagnosis as well as in the understanding of the metabolic changes which cancer cells are subjected to within their microenvironment [18].

Some studies have found modifications in several lipid families in BC patients compared to the control population and correlated these alterations with a higher frequency of cancer occurrence [19]. Furthermore, it has been reported that there is an increase in levels of phospholipids, phosphocholines, cholines and glycerophosphocholines in tumors [20,21]. However, it is also important to note that plasma contains a high variety of lipids that can be altered according to dietary sources [22].

In order to understand the metabolic switch that tumor cells undergo within their microenvironment, we performed in-depth lipid profiling of four different BCC lines, MDA-MB-231, BT474, SKBR3, and MCF-7 as well as the MCF-7 derived drug resistant MCF-7 TAX^R^ and MCF-7 Epi^R^ cells in close contact with mature adipocytes. Our main objective was to understand how the adipose tissue within the tumor microenvironment transfer lipids to adjacent cancer cells and how the tumor lipid metabolism is modified as a result, using lipidomics techniques and analysis.

Previous studies have detected a different lipidic and lipoprotein profile in breast cancer patients compared to healthy women [23]. Knowledge of a specific lipid signature and a particular lipid and lipoprotein profile might help in breast cancer diagnosis and in the improvement of new possible therapies.

## 2. Materials and Methods

### 2.1. Cell Culture

Human MDA-MB-231, BT474, SKBR3, MCF-7 and its resistant MCF-7 TAX^R^ and MCF-7 Epi^R^ were described previously and originally obtained from the American Type Culture Collection (ATCC). Cell lines were cultured Dulbecco’s modified Eagle’s medium (DMEM) supplemented with 10% foetal bovine serum (FBS), 1% penicillin streptomycin and 1% non-essential amino acids (Biowest, Barcelona, Spain) at 37 °C with 5% CO_2_.

Fibroblast cell line 3T3-L1 was obtained from the American Type Culture Collection (ATCC) and it was cultured until confluence. Then, they were cultured for 10 days with differentiation media, consisting in DMEM supplemented with 10% FBS, 1% penicillin/streptomycin, 1% Non-essential Amino Acid Solution, 0.5 mM 3-isobutyl-1-methylxanthine (IBMX), 1 µM dexamethasone and 10 µg/mL insulin from bovine pancreas (Sigma, Barcelona, Spain).

After differentiation, mature adipocytes were serum starved for 24 h. Then, they were cultured with the necessary conditions for each experiment.

### 2.2. Conditioned Media Obtainment

To obtain conditioned media (CM), MDA-MB-231, BT474, SKBR3, MCF-7 and its resistant MCF-7 TAX^R^ and MCF-7 Epi^R^ were cultured until they reached 80% of confluence. Then cells were serum starved. After 24 h of incubation with 0.1% FBS, different BCC lines CM were obtained for further assays. Moreover, mature adipocytes were cultured for 3 days with the different BCC lines CM. As a control condition, mature adipocytes were serum starved for 3 days.

### 2.3. Oil Red Staining

Oil Red O is a dye which strongly stains neutral lipids. To determinate the adipocyte lipid content, mature adipocytes were cultured with different BCC lines CM. For 3 days of incubation, treated and non-treated mature adipocytes were stained with Oil Red dye following the protocol described (#K580-24) and pictures were taken with an inverted microscope (Olympus IX71). Then, three washes with isopropanol 60% were performed and for dye extraction, cells were incubated with isopropanol 100% for 5 min. Afterwards absorbance at 490 nm was measured using SINERGY HT (BioTek, Swindon, UK).

### 2.4. RNA Extraction and Quantitative Real Time (qRT) PCR

RNA extraction was performed according to the PureLink RNA Mini Kit (Invitrogen, ThermoFisher Scientific, Barcelona, Spain) protocol and it was quantified using Synergy HT (BioTek, Swindon, UK). Total RNA (1 μg) was reverse-transcribed using the PrimeScript RT Reagent Kit (Takara Bio, Saint-Germain-en-Laye, France). Levels of mRNA were assessed using LightCycler96 device (Roche) with the Taqman probes for respective genes acquired from Life technologies CD36 (Mm00432403_m1), FABP4 (Mm00445878_m1), FABP5 (Mm00783731_s1), TBP (Mm01277042_m1). Then, transcript results were normalized with the gene TBP and the fold change was obtained.

### 2.5. Bodipy Fluorescent Staining

To observe the lipid transfer from mature adipocytes to BCC lines MDA-MB-231, BT474, SKBR3, MCF-7 and its resistant MCF-7 TAX^R^ and MCF-7 Epi^R^, fibroblasts were differentiated for 10 days as previously described. During the differentiation process labeled palmitic-acid BODIPY (Sigma) every other day. In order to test the intake of labeled palmitic acid during the differentiation process, different images were taken using an inverted microscopy (Olympus IX71) and fluorescent intensity measure by ImageJ.

Alternatively, after maturation, the labeled adipocytes were serum-deprived for 3 days, and the medium was collected. Then, the BCC lines to be examined were seeded into 24 well plates until they reached 80% confluence and they were serum-starved with DMEM high glucose 0.1% FBS for 24 h. Next, the medium derived from labeled adipocytes were added to the BCC lines. After 72 h of treatment, cells were washed with PBS 1X, and images were taken using an inverted microscopy (Optimus IX71).

### 2.6. Enzyme-Linked Immunosorbent Assay (ELISA)

In order to determine the protein levels from adipocytes CM, ELISA assay was performed to detect the levels of FABP4 and CD36. The media from the adipocytes and the CAAs were obtained as explained above. The samples were analyzed performing ELISA assay following the company protocols (Antibodies Online).

### 2.7. Lipid Uptake Assay: Nile Red Staining

Different BCC lines were seeded in 12 well plates. After serum-starvation, cells were incubated with adipocyte CM and they were stained after 24 h with the lipophilic fluorescent dye Nile Red (100 ng/mL) (Sigma-Aldrich; Barcelona, Spain) diluted in PBS 1× for 5 min at room temperature to visualize the lipid droplets. Cell images were captured using an Olympus IX71 microscope and analyzed using the Image J software obtaining the intensity of each image.

### 2.8. C^13^ Palmitic Assay

To assess the lipid transfer from mature adipocytes to the different BCC lines, a coculture system was performed. Firstly, 3T3-L1 fibroblasts were seeded and cultured in the upper chamber of a 12-well plate until they reached confluence. Then, they were differentiated as described above. Every other differentiation day, an isotope of palmitic acid (PA) with C^13^ was added. For PA formulation, C^13^PA (200 µM) was mixed with free fatty acid BSA (33.32 µM) (Sigma). As a control condition, a parallel differentiation with non-isotope PA was performed. 

In a 12 well plate, different BCC lines were seeded and cultured until they reached 80% of confluence. Then, the upper chamber with mature labeled and non-labeled adipocytes were put on above these BCCs. After 2 days of coculture, cells were harvested and collected until their analysis.

### 2.9. Lipidomics

Cells and culture media lipids were extracted using a biphasic extraction method. Cells were extracted in 220 µL methanol. After sample fragmentation by vortexing, immersion in liquid N2, and ultrasonication, 440 µL of dichloromethane (DCM) and 140 µL of water were added sequentially. A total of 200 µL of media was extracted via the same procedure as the cells without the immersion in liquid N2 and using methyl tert-butyl ether (MTBE) instead of DCM. Samples were incubated at 4 °C for 30 min and centrifuged (at 15,000 rpm for 15 min at 4 °C). 330 µL of the organic phase (lipidic) was collected for drying under a stream of nitrogen. Lipid pellets were resuspended in 150 µL of methanol/toluene (9:1) for liquid chromatography–mass spectrometry (LC–MS) analysis. Quality control (QC) samples consisting of pooled samples from each condition were injected at the beginning and periodically through the workflow.

Untargeted LC–MS analyses were performed using a UHPLC system (1200 series, Agilent Technologies) coupled with a 6550 ESI-QTOF MS (Agilent Technologies) operating in positive electrospray ionization (ESI+) mode. A total of 2 µL of cells extract and 3 µL of media extract were injected, and lipids were separated by reverse-phase chromatography with an Acquity UPLC C18-RP (ACQUITY UPLC BEH C18 1.7 µM, Waters). Mobile phase A was acetonitrile/water (60:40) (10 mM ammonium formate), and mobile phase B was isopropanol/acetonitrile (90:10) (10 mM ammonium formate). Solvent modifiers were used to enhance ionization and to improve the LC resolution in positive ionization mode. Separation was conducted under the following gradient: 0–2 min, 15–30% B; 2–2.5 min, 48% B; 2.5–11 min, 82% B; 11–11.5 min, 99% B; 11.5–12 min, 99% B; 12–12.1 min, 15% B; 12.1–15 min, 15% B. The ESI conditions were as follows: capillary voltage, 4000; gas temperature, 150 °C; drying gas, 12 L min^–1^; nebulizer, 30 psig; fragmentor, 120 V; and skimmer, 65 V. The instrument was set to work over the m/z range from 50 to 1200 with an acquisition rate of 3 spectra/sec. For compound identification, MS/MS analyses were performed in targeted mode with an acquisition rate of 3 spectra/sec, applying three collision energies:10, 20, 30, and 40 V. Lipid structures were identified by matching tandem MS spectra against reference standards in LIPID MAPS [24] and/or LipidBlast [25] and/or Metlin [26].

LC–MS data were processed using the XCMS R package [2] for peak picking, retention time alignment and feature detection. Then, stable isotopic labelling was detected using geoRge R [27] package in order to find ^13^C enriched lipid metabolites. This analysis provided a matrix containing the retention time, ion m/z value, number of ^13^C enriched and the integrated the peak area of each feature for each sample. Features were then putatively associated to lipid identities found in above mentioned databases. Only those annotated features were statistically tested for significant changes across experimental groups using a one-way ANOVA, correcting for multiple comparisons using Tukey’s honest significant differences and false discovery rate (FDR) method.

### 2.10. Statistical Analysis

All analyses were performed with the Statistical Package for Social Sciences (SPSS) software 27.0.1.0 (Madrid, Spain). Data were expressed as mean ± SEM. Statistical analysis was determined by one-way ANOVA and the differences between groups was analyzed using the Tukey post-hoc test. A *p*-value < 0.05 was considered to be statistically significant.

## 3. Results

### 3.1. Breast Cancer Cell Conditioned Media Enhances Delipidation of Mature Adipocytes

To assess if tumor cells can influence the amount of lipids liberated by the adipocytes, mature adipocytes were cultured with conditioned media from different BCC lines (MDA-MB-231, SKBR3, BT474, MCF-7 and the resistant MCF-7 EPI^R^ and MCF-7 TAX^R^) and the amount of lipids accumulated in the mature adipocytes was then examined using Oil Red staining.

**Figure 1:**
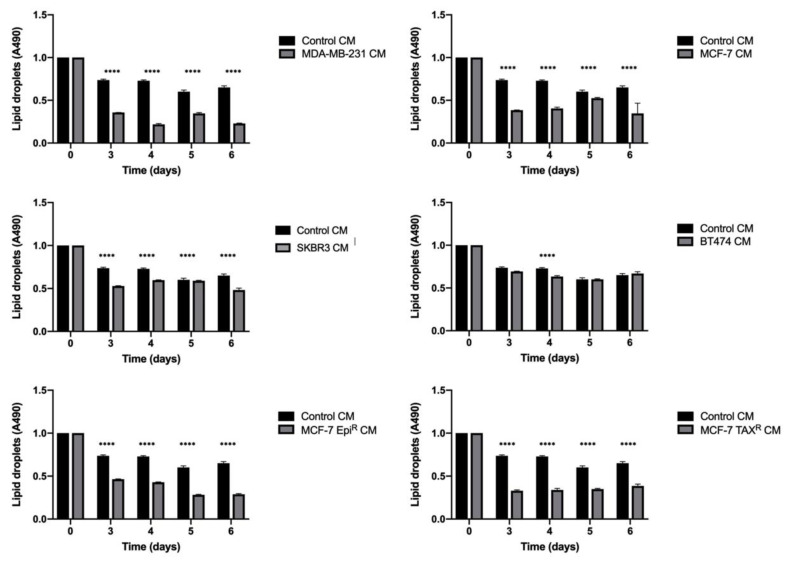
BCC CM increases mature adipocyte delipidation. Graph illustration of Oil Red dye in mature adipocytes at different conditions of culture for 6 days. The Oil Red tinction is represented according to colorimetric signal that is measured at an absorbance of 490 nm and symbolizes the amount and size of lipid droplets within mature adipocytes. In the graphic, black bars represent the control condition, while grey bars indicate those conditions where mature adipocytes have been cultured with a different BCC line CM. There is a significant decrease in Oil Red dye within those adipocytes that have been cultured with all the different BCC lines CM compared to those adipocytes cultured in control conditions. One-way ANOVA where a *p* < 0.05 was considered statistically significant (**** < 0.0001).

Interestingly, we observed that incubation with CMs from BCC lines caused the intracellular lipid droplet levels to decrease drastically when compared to the adipocytes incubated with the control CM (Figure 1). Accordingly, over 3 days of culture, the lipid levels of adipocytes incubated with BCC CMs declined significantly when compared with the control adipocytes (all CMs: *p* < 0.001; one-way ANOVA test).

### 3.2. Breast Cancer Cell Conditioned Media Enhance FABP4, FABP5 and CD36 Release from Mature Adipocytes

**Figure 2:**
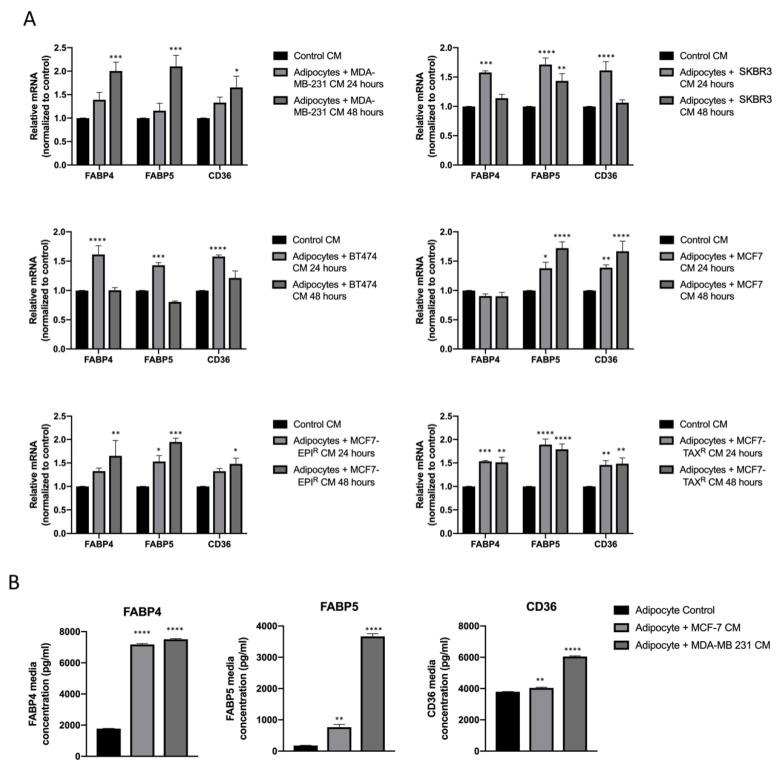
BCC CM enhances FABP4, FABP5 and CD36 mRNA levels and increases their releasement to media. (**A**)Graph representation of FABP4, FABP5 and CD36 transcripts in mature adipocytes at different conditions for 24 and 48 h of treatment. There is a clear and significant increase of all three transcripts in those mature adipocytes that have been culture with BCC line CM. Interestingly, FABP4 transcript in mature adipocytes culture with MCF-7 CM is not affected unlike the rest of conditions and transcripts. One-way ANOVA where a *p* < 0.05 was considered statistically significant. (**B**) Media from control adipocytes is compared with media from adipocytes cultured with MDA-MB-231 and MCF-7 cell lines conditioned media, for 24 h where a *p*-value < 0.05 is statistically significant (* < 0.05, ** < 0.005, *** < 0.0005, **** < 0.0001).

Next, we explored the mechanism responsible for higher levels of fatty acid export and examined the expression levels of adipocyte-expressing fatty acid transporter proteins (i.e., FABP4, FABP5 and CD36) in the mature adipocytes upon culture with BCC CMs by RT-qPCR. The results showed that the mRNA levels of FABP4 and CD36 were significantly increased in the adipocytes when culture with BCC CMs (* *p* < 0.05) one-way ANOVA) (Figure 2A).

In addition, once we analyzed two of the most representative BCC lines (MDA-MB-231 and MCF-7) effects on adipocyte CM composition, we observed that there was an increase of all three fatty acid transporters within the media (Figure 2B). BCC lines MDA-MB-231 and MCF-7 CM produces an increase in fatty acid transporters mRNA in mature adipocytes, but also increases the releasement pattern of these fatty acid transporter proteins (* *p* < 0.05) one-way ANOVA.

### 3.3. Mature Adipocytes Conditioned Media Increases Fatty Acid Uptake in Breast Cancer Cell Lines

**Figure 3:**
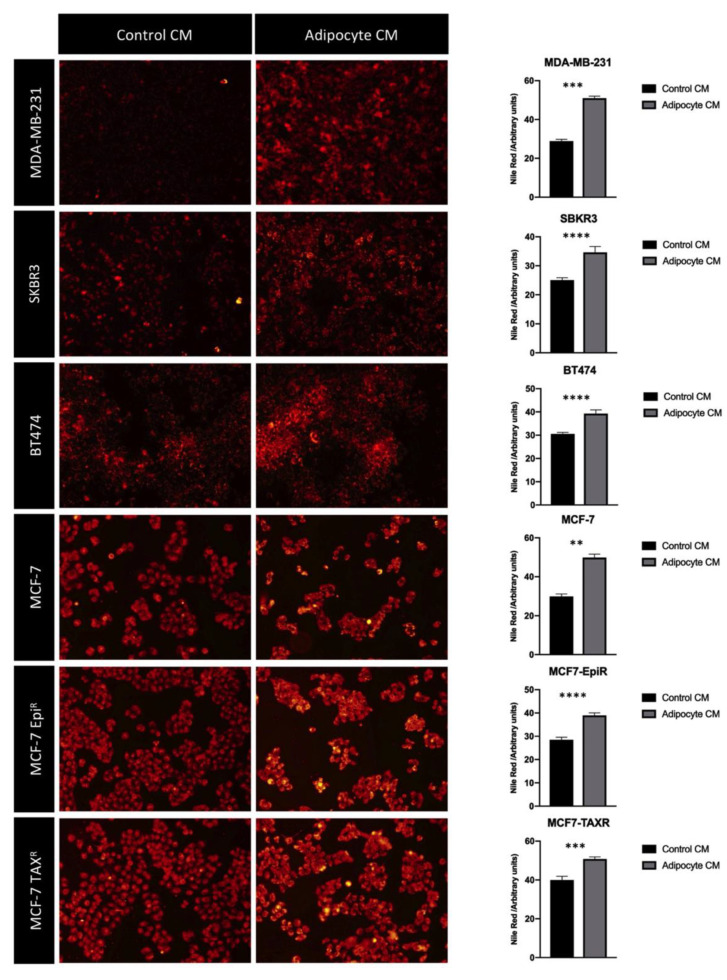
Mature adipocyte CM increases lipid uptake in BCC lines. Immunofluorescence images (10× magnification) of different BCC lines stained for fatty acids using Nile Red stain. There is an increase in Nile Red staining once cells were cultured with adipocyte CM compared to those cells cultured in control conditions. Graphs represent Nile Red staining intensity which could be translate to the amount of fatty acid taken up by the cancerous cells. One-way ANOVA where a *p* < 0.05 was considered statistically significant (** < 0.005, *** < 0.0005, **** < 0.0001).

Lipids are an important source of nutrients for the tumor cells, and they can be oxidized in order to generate energy and support growth. We have found a higher degree of delipidation and an increase in fatty acid transporter expression in mature adipocytes when cultured with BBC conditioned media. We hypothesized that those lipids secreted by the adipocytes are taken up by the BCCs. To test this conjecture, we cultured the BCC lines MDA-MB-231, SKBR3, BT474, MCF-7, MCF-7-EPI^R^ and MCF-7-TAX^R^ with the control or adipocyte CM and studied their lipid uptake by staining their intracellular accumulated lipid droplets using Nile Red. The results showed that there was a significant increase in the amount of lipid droplets in all the BCC lines following incubation with adipocyte CM when compared with control CM (*p* < 0.05; one-way ANOVA) (Figure 3).

### 3.4. Mature Adipocytes Internalize Palmitic Acid during Differentiation and Export it as Lipids to Breast Cancer Cells

**Figure 4:**
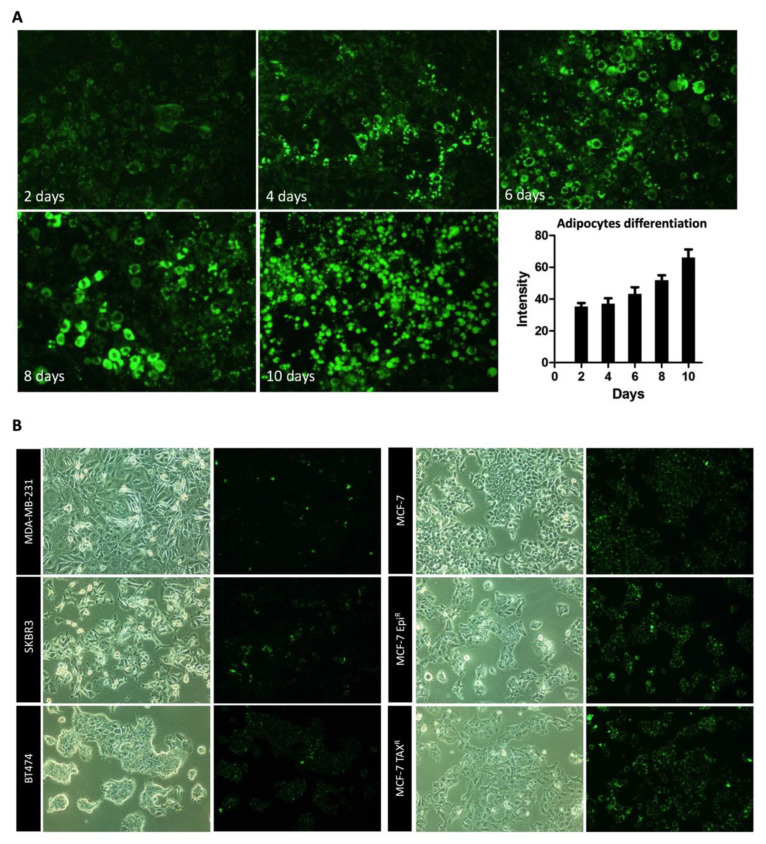
BCCs internalize palmitic acid from mature adipocytes. (**A**) Immunofluorescence images (10× magnification) of the fibroblasts 3T3-L1 cell line during their differentiation process for the labeled palmitic acid (BODIPY). Graph represents the BODIPY intensity within the preadipocytes during their differentiation until they reached the complete process. (**B**) Immunofluorescence images (10× magnification) of different BCC lines for BODIPY. Once it was obtained complete differentiated adipocytes with BODIPY incorporated, they were serum starved and adipocyte CM was obtained. Then, the different studied BCC lines were cultured with this adipocyte CM, and it was tested if there was BODIPY label within the cancerous cells. As it is represented in the pictures, all the BCC lines were able to uptake BODIPY from media within their cytoplasm.

Palmitic acid is a fatty acid closely linked to cancer progression [28]. We sought to confirm if lipid droplets accumulated in the BCCs are indeed derived from the uptake of lipids released from the adipocytes. To this end, we first performed a negative and positive control to show a specific PA-BODIPY labelling (Figure S1). After that, we differentiated the murine fibroblast 3T3-L1 cells into adipocytes in the presence of the fluorescently labeled palmitic acid (BODIPY) and measured the fluorescent signal during the fibroblast differentiation into mature adipocytes. The result showed that the amount of lipid droplets increased as along with an increase in fluorescent BODIPY signal during adipocyte differentiation (Figure 4A).

Then, we assessed if the lipids accumulated in the mature adipocytes, are released by the adipocytes and uptaken to the different BCC lines. We analyzed the levels of intracellular BODIPY fluorescence in the BCCs after cultured them with PA-labeled adipocyte CM and found a substantial increase in labeled palmitic acid in the cytoplasm of BCCs, suggesting that the accumulation of lipids in the BCCs when co-cultured with adipocytes as a result of their uptake of lipids released by the adipocytes (Figure 4B).

### 3.5. Breast Cancer Cells have a Different and Specific Lipid Signature in the Palmitic Transformation and Resistant Cells Lipid Pattern Differs from Their Sensitive Cell Line Being Closely to TNBC Cell Line Pattern

Next, we sought to identify the specific changes in lipid compositions in the breast cancer cells in response to co-culture with adipocytes. ^13^C labeled palmitic acid was added to differentiating adipocyte, which were then co-cultured with different BCCs, respectively. Further mass spectrometry analysis revealed that the ^13^C labeled PA had transformed into different lipid species in the BCCs. The results showed that there were new lipid species, such as DAG, TG, ceramides, phosphatidylcholines, phosphoethanolamines and sphingomyelins, whose carbons were derived from the mature adipocytes derived ^13^C-labeled PA. Once we analyzed the different BCC lines one by one, we observed that some BCCs had a similar but specific lipid pattern that were distinct from other BCCs. For example, we uncovered that SKBR3 and BT474 cells had a similar lipid signature. Through principal component analysis, we found that both SKBR3 and BT474 cells were closely related in terms of their lipid metabolism, particularly the way they convert the labeled fatty acids into DAGs and SMs. Moreover, we also detected that their lipid species, including diacylglycerols (DAGs), sphingomyelins (SMs), triglycerides (TGs) and phosphatidylcholines (PCs), are notably different from the other BCC lines studied. When we compared the luminal A MCF-7 cell line with its resistant derivatives, MCF-7 EPI^R^ and MCF-7 TAX^R,^ through principal component analysis, we observed that they were remarkably different in their lipid metabolic pathways by which the labeled fatty acids were transformed into intracellular lipids. Amongst the lipids which differed substantially between the parental and drug-resistant MCF-7 cells were ceramides (CMs), sphingomyelins (SMs), phosphatidylethanolamines (PEs) and phosphatidylcholines (PCs).

**Figure 5:**
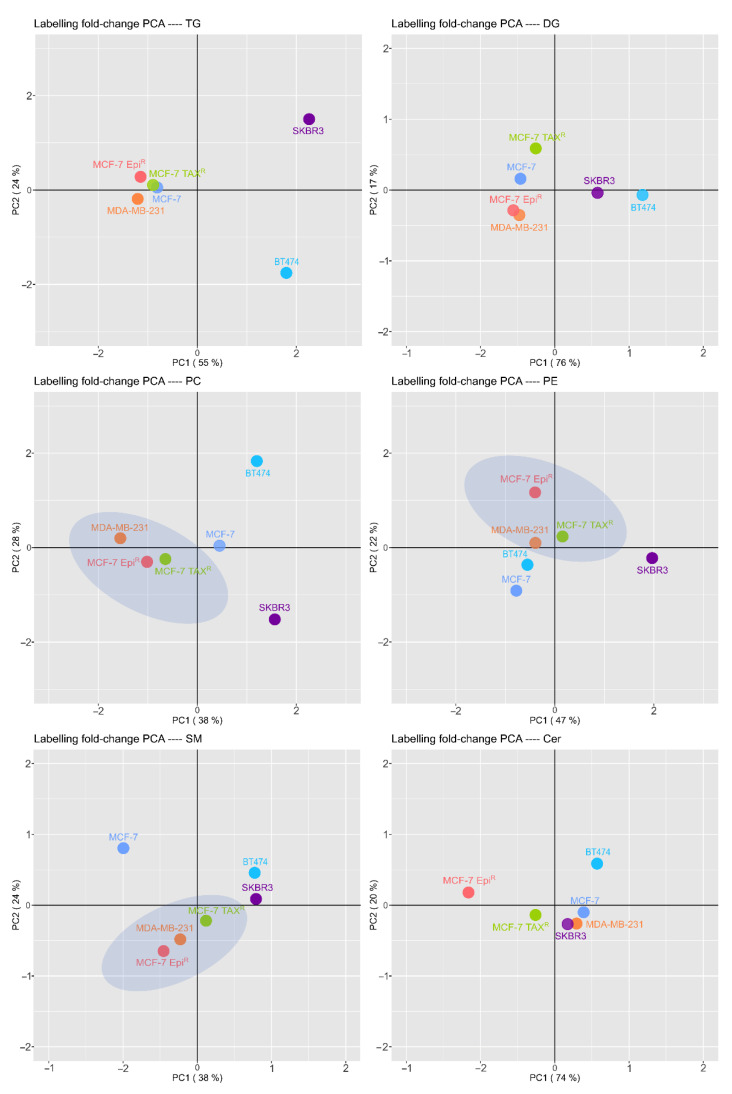
BCCs have a specific lipid signature that differs from each cell line. Principal component analysis (PCA) score plots of the different BCC lines analyzed by LC-MS. Scrutiny of the analyzed BCC lines indicated the similarities and differences between the different lipid species (DG, TG, PC, PE, SM and Cer). It is clear a differential lipid pattern between the studied BCC lines, where the luminal B cell line BT474 and the HER2+ cell line SKBR3 are notably differentiated in their lipid pattern from the rest of cell lines. Moreover, they are notably separated between themselves. Therefore, analyzing the lipid patterns of the BCC lines MCF-7 and its resistant cell lines, MCF-7 Epi^R^ and MCF-7 TAX^R^, there were significant differences on them. In addition, resistant BCC lines lipid patterns were more similar to the lipid pattern of TNBC line MDA-MB-231 than the lipid profile of sensitive cell line MCF-7 (DG: diacylglycerol, TG: triacylglycerol, PC: phosphatidylcholine, PE: phosphatidylethanolamines, SM: sphingomyelin, Cer: ceramide).

Surprisingly, the principal component analysis also showed that drug resistant MCF-7 TAX^R^ and MCF-7 EPI^R^ cells harbored a lipid signature more similar to the triple negative breast cancer (TNBC) cell line MDA-MB-231. (Figure 5).

Subsequently, the lipid families were also analyzed individually between different breast cancer cell lines. The principal component analysis also revealed a specific lipid metabolic pattern that is shared between the drug resistant MCF-7 cells and the TNBC MDA-MB-231 cell lines, which is distinct from the parental MCF-7 cells. In-depth individual lipid family analysis revealed that phosphatidylcholines (PCs), such as PC(O-33:3), PC(38:1) and PC(20:0/18:1) were increased in the drug-resistant MCF-7 and TNBC cells compared to the other drug-sensitive cell lines. Among the lipids, the phosphatidylethanolamines, including PE(P-34:2)///PE(P-16:1/18:1), PE(P-36:2)///PE(P-18:1/18:1), PE(O-34:2)///PE(O-15:1/19:1), PE(P-38:7)///PE(P-16:1/22:6), the ceramides (CMs), such as LacCer(18:1/16:0) and LacCer(18:1/24:0) and the sphingomyelins (SMs), such as SM(36:1) and SM(42:1), were also altered in both the drug resistant MCF-7 cells and MDA-MB-231 cells when compared with the rest of the drug-sensitive cell lines (Table 1).

In summary, these alterations demonstrate a switch in lipid components and metabolism in the drug resistant MCF-7 Epi^R^ and MCF-7 TAX^R^ and the MDA-MB-231 cell lines when compared with the other relatively more drug-sensitive cell lines. Moreover, as MDA-MB-231 cells have previously been shown to be a more taxol and epirubicin resistant cell line compared to MCF-7 and some of the other breast cancer cell lines studied, our results also suggest that the more drug-resistant and malignant breast cancer cells have modified their lipid profiles and metabolism to enhance their drug-resistance and malignant progression.

## 4. Discussion

The interactions between different components of the tumor microenvironment and the cancer cells play a key role in cancer initiation and malignant progression [6]. In consequence, their cross-talks have been the objects of cancer research during last decades, and huge advances have been achieved in deciphering these interactions as well as the mechanistic details involved. One of the major components of the breast is adipose tissue; hence, the cross-communication between breast cancer cells and their nearby adipocytes play a crucial part in breast cancer progression. Different mechanisms for interactions between the breast cancer cells and the adipocytes have been suggested, but the lipid transfer from adipocytes to cancerous cells have gained special attention. In fact, earlier studies have already established a close relationship between lipid transfer from adipocytes to breast cancer cells and the enhancement of several well-established cancer hallmarks in breast cancer. However, the specific mechanisms that underly these cross-communications between adipocytes and breast cancer cells are yet to be unveiled.

In this study, we also studied the interactions between adipocytes and six different breast cancer cell lines, including MDA-MB-231, SKBR3, BT474, MCF-7 and the MCF-7 derived drug-resistant cell lines MCF-7 Epi^R^ and MCF-7 TAX^R^. The results have expanded our understanding of the effects of many types of breast cancer cells, including TNBC, HER2+, luminal A and luminal B as well as drug-resistant breast cancer cells on mature adipocytes and vice versa in the tumor microenvironment.

In this study, we firstly focused on how tumor cells modify the tumor microenvironment, focusing on the adipose tissue. We reviewed how different breast cancer cells modify the behavior of adipocytes within the tumor microenvironment and reconfirmed the previous finding that breast cancer cells promote lipid metabolism and increase its degree of delipidation [11]. We also found that breast cancer cells induce the transcription of different fatty acid transporters such as FABP4, FABP5 and CD36. We also obtained evidence that these fatty acid transporter proteins are also released with the lipids by the adipocytes into their surroundings, suggesting that their release might promote the transport and metabolism of fatty acids in the breast cancer cells. In agreement, these fatty acid transporter proteins have been intimately related to cancer development and malignant progression [29,30].

Moreover, it has also been suggested that tumor cells cocultured with mature adipocytes increases their lipid uptake, which is partially mediated by the fatty acid transporters [13,31,32].

Recently, palmitic acid has been shown to be strongly linked to cancer development and progression. In fact, it has been demonstrated tumor cells have a specific need for this lipid and it is capable of promoting a number of cancer hallmarks, particularly cancer survival and metastasis [33]. It has also been also demonstrated that there is a strong positive correlation between palmitic acid level and tumor progression [28].

As a result, we tracked the labeled palmitic acid, and confirmed that mature adipocytes first incorporated the labeled fatty acid, and then released it into the extracellular media, when they are treated with the conditional media from different breast cancer cell lines. We also obtained evidence that adipocyte-released fatty acids were taken up by the breast cancer cells and turned into lipids. Moreover, the internalized fatty acids will be used for the biosynthesis of new lipid compounds in the breast cancer cells, and the discovery of specific lipid signatures and metabolic pathways for distinct molecular breast cancer subtypes may help us to understand how different cancer cells internalize lipids from adjacent adipocytes and how they transform them into any lipid families in their own benefit. In fact, a previous study has demonstrated that cancer cells incorporate exogenous palmitic acid into their structural and oncogenic signaling lipid components [34]. It has been shown that fatty acid uptake and lipid metabolism promote cancer progression and metastasis.

To determine the lipid metabolic pathways involved in enhancing cancer progression and metastasis, we first cultured the fibroblast cell line 3T3-L1 in the presence of ^13^C-palmitic acid and confirmed that this isotope-labeled fatty acid was internalized into mature adipocytes as lipid droplets. Then, the adipocytes carrying the isotope-labeled fatty acid were cocultured with our different breast cancer cell lines. We observed a significant increase of the isotopic labels in media, suggesting that there was a releasement of these lipids form mature adipocytes to their environment. Subsequently, we also observed that the isotope signals that had appeared in media disappeared slowly into the cancer cells, suggesting the labeled lipid internalization into the cancer cells.

Next, we analyzed the fate of the labeled palmitic acid carbon in all different breast cancer cell lines using a LC-MS analysis, and interestingly, we found that cancer cells were able to synthetize new lipid species.

As cancer cells need a huge amount of lipids in order to proliferate, to synthesize cellular structural components and to participate in intracellular signaling [5].

Phosphatidylcholines are one of the main lipid subtypes present in the mammalian cells and their metabolism has been widely studied in cancer. According to bibliography, it has been described that there is an increase in the presence of different PC in worst prognosis BC patients’ blood stream [20]. Moreover, there is high correlation between lipid metabolism, including PC (14:0/16:0) for example, and the tumor grade [35].

Our results show significant differences between the PC pattern of each studied BCC line. Interestingly, the PC biosynthesis pattern was more similar between the resistant MCF-7 cell lines MCF-7 Epi^R^ and MCF-7 TAX^R^ and the TNBC cell line MDA-MB-231 compared to the sensitive cell lines MCF-7, the luminal B cell line BT474, and the HER2+ cell line SKBR3. Interestingly, PC saturations such as PC (36:1), PC (38:2) have been closely related with worst prognosis [36,37].As mentioned earlier, similar to MCF-7 Epi^R^ and MCF-7 TAX^R^ cells, MDA-MB-231 cells have previously been shown to be a more taxane (e.g., taxol) and anthracyclin (e.g., epirubicin) resistant cell line and more metastatic competence compared to MCF-7 and some of the other breast cancer cell lines used in the study [38]. Our results therefore suggest that the more drug-resistant and malignant breast cancer cells have modified their lipid profiles and metabolism to enhance their drug-resistance, metastatic competence and malignant progression.

We have found that the way that the drug resistant MCF-7 Epi^R^ and MCF-7 TAX^R^ cells and the TNBC MDA-MB-231 cells metabolize the palmitic acid derived from mature adipocytes in a different manner, in comparison to the parental drug-sensitive MCF-7 as well as to the luminal B and HER2+ cell lines, suggesting that more malignant and drug-resistant breast cancer cells acquire a specific PC biosynthesis pattern that differ from the drug-sensitive and less metastatic breast cancer cells. Furthermore, alterations in PE metabolism have been also linked with breast cancer progression. In fact, a number of studies have reported altered plasma and urine PE levels, specifically PE (15:0/19:1) to be linked to breast cancer development. In this study, we have also found this lipid family strongly altered in the drug-resistant cell lines (i.e., MCF-7 Epi^R^, MCF-7 TAX^R^ and MDA-MB-231 cells) compared to the relatively more drug-sensitive cell lines.

Bioactive sphingolipids and ceramides are of special importance in promoting cancer progression due to their roles as signaling molecules and have been shown to regulate a number of critical cellular biological processes [10,39]. SM is one of the most abundant sphingolipids in normal cells and deregulated expression and function of this lipid family have also been correlated with breast cancer development and progression [36]. According to our results, we also detected an alteration in the signature of this family of lipids in the relatively more resistant cell lines. In our study, a similar signature of ceramide expression was also found in the drug-resistant breast cancer cells. Ceramides constitute the structural scaffold of sphingolipids and it has been described that metabolites derived from ceramides are involved in the signal transduction of a number of signaling pathways involved in apoptosis inhibition and cell growth promotion [40,41]. In addition, high levels of ceramide synthase and ceramide has been found in breast cancer compared to normal non-cancerous tissues [42].

## 5. Conclusions

**Figure 6:**
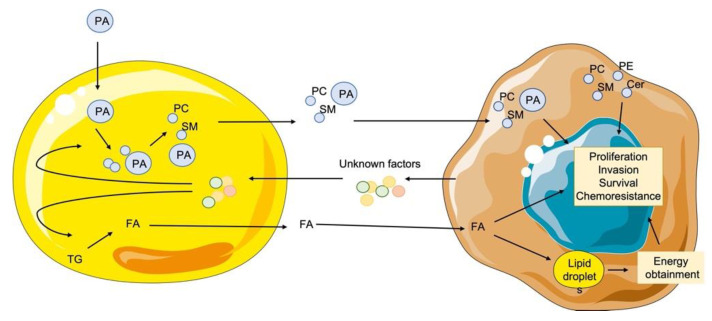
Schematic representation of the crosstalk between adipocytes and breast cancer cells. Palmitic acid (PA) is internalized into mature adipocytes during their differentiation. Then the coculture of both cell lines produces a releasement of unknown factors from cancerous cells that modifies the behavior of adjacent adipocytes increasing their catabolism, thus enhancing their delipidation. In addition, PA is metabolized, and new species are formed from this lipid, such as phosphatidylcholines (PC) or sphingomyelins (SM). Close contact between both cell types leads to the releasement of these lipid species, being internalized into the cancerous cells. Then, new lipidic species are produced form these lipids, including phosphatidylethanolamines (PE) and ceramides (Cer). The specific lipid pattern will confer an enhancement in the tumorigenic behavior on the breast cancer cells. In addition, free fatty acids also are released form adjacent adipocytes and are internalized into the cancerous cells. There, they are included into lipid droplets that will confer a new source of energy, although they also can induce the enhancement of the cancer cells hallmarks.

In summary, our study shows that defining the lipid signatures in breast cancer might be a new approach to understanding the behavior of cancer cells within the tumor microenvironment, particularly when they are in the vicinity of the adipose tissue. Moreover, our data also suggest that altered lipid metabolism may play a critical role in the malignant transformation as well as drug-resistance in the breast cancer cells. The altered lipid signatures found in the drug-resistant and more metastatic breast cancer cells might also be useful markers for breast cancer diagnosis and prognosis. Moreover, targeting the deregulated lipid uptake and metabolism in the drug resistant breast cancer cells may help to reverse the drug insensitivity of some breast cancer cells and lead to novel treatment strategies for breast cancer as well as drug resistance (Figure 6).

## Figures and Tables

**Table 1:** Resistant variants of MCF-7 have a lipid signature more similar to TNBC. Presence of different lipid families such as PC, PE, TG, SM and Ceramides in resistant cell lines MCF-7 Epi^R^ and MCF-7 TAX^R^ and the TNBC cell line MDA-MB-231 compared to the presence that is detected in the sensitive cell line MCF-7. It is described the fold change of each specific lipid within each family mentioned above that has been found within these four cell lines. There are differences in the presence of many PC, PE, SM and Cer, where an increased fold increase is present once their presence is compared between resistant and TNBC lines compared to sensitive cell line MCF-7 (green underlined).

Lipid Species		MCF-7 Epi^R^ vs. MCF-7		MCF-7 TAX^R^ vs. MCF-7		MDA-MB-231 vs. MCF-7	
		Fold Change	*p* Value	Fold Change	*p* Value	Fold Change	*p* Value
**PC**	**PC(28:0)///PC(14:0/14:0)**	0.02990952	**0.001**	0.04615794	**0.001**	0.21447702	0.050
	**PC(31:1)///PC(15:0/16:1)**	0.0689704	**0.017**	0.09403492	**0.025**	0.20846594	0.108
	**PC(30:1)///PC(16:1/14:0)**	0.01112429	**0.002**	0.00700047	**0.000**	0.01721311	**0.002**
	**PC(O-33:3)**	1.27651749	**0.004**	3.99971727	**0.001**	2.80090791	**<0.001**
	**PC(38:3)///PC(18:0/20:3)**	0.27554774	**0.044**	0.26114733	**0.007**	0.65273596	0.738
	**PC(44:1)///PC(26:0/18:1)**	44.6634837	**0.092**	36.8772782	**0.032**	376.01122	**0.001**
	**PC(38:1)///PC(20:0/18:1)**	3.53236174	0.288	3.43871497	0.184	14.0520945	**0.004**
**PE**	**PE(P-34:2)///PE(P-16:1/18:1)**	28.2972788	**<0.001**	26.2359375	**<0.001**	22.5278479	**<0.001**
	**PE(P-36:5)///PE(P-16:1/20:4)**	8.81558854	**0.007**	10.4244025	**0.005**	14.2692477	**0.001**
	**PE(P-36:2)///PE(P-18:1/18:1)**	25.9952287	**<0.001**	19.896668	**<0.001**	114.690505	**<0.001**
	**PE(34:1)///PE(16:0/18:1)**	0.58971808	0.781	0.64063147	0.186	0.35193966	**0.009**
	**PE(O-34:2)///PE(O-15:1/19:1)**	29.4014166	**<0.001**	23.5110141	**<0.001**	22.0164115	**<0.001**
	**PE(P-38:7)///PE(P-16:1/22:6)**	14.7720082	**0.006**	12.4773801	**0.004**	9.72151443	**0.008**
**TG**	**Mix TG(44:1)**	0.03544991	0.003	0.04252924	**0.001**	0.04335925	**0.001**
	**Mix TG(46:4)**	0.05574601	**<0.001**	0.07728431	**<0.001**	0.07396131	**<0.001**
	**Mix TG(46:1)**	0.15810058	0.018	0.23540369	**0.004**	0.17216579	**0.002**
	**Mix TG(48:4)**	0.29777056	0.007	0.40895724	**0.008**	0.27500008	**0.001**
	**Mix TG(50:5)**	0.16080286	**<0.001**	0.17598899	**<0.001**	0.12880096	**<0.001**
	**Mix TG(50:2)**	0.48675871	0.399	0.53214893	**0.017**	0.39973327	**0.002**
	**Mix TG(52:3)**	0.24951721	0.092	0.30606399	**0.011**	0.34407207	**0.013**
	**Mix TG(54:3)**	0.47035591	0.322	0.38418791	**0.001**	0.40637479	**0.002**
**SM**	**SM(36:1)**	3.3947483	**0.001**	3.89343315	**0.002**	1.02089675	0.999
	**SM(39:3)**	0.22395786	**0.017**	0.21373755	**0.001**	0.21085366	**0.002**
	**SM(42:2)**	0.55261516	0.632	0.37982867	**0.013**	0.46507858	0.086
	**SM(42:1)**	2.07385942	0.331	2.05459388	0.309	5.87791467	**0.003**
**Cer**	**LacCer(18:1/16:0)**	57.8502342	**<0.001**	48.320848	**<0.001**	4.02257761	**0.003**
	**DihydroCer(18:0(OH)/16:0)**	0.20022379	0.073	0.18622892	**0.003**	0.10548975	**0.001**
	**LacCer(18:1/24:0)**	103.862204	**<0.001**	56.4934583	**<0.001**	10.0750913	**<0.001**

## Data Availability

The data presented in this study are available on request from the corresponding author.

## Supplementary Materials

The following are available online at https://www.mdpi.com/article/10.3390/cancers13235871/s1, Figure S1: Negative and positive control of PA-BODIPY labelling in BCC.
