# Supplementary material for: MCF-7 Drug Resistant Cell Lines Switch Their Lipid Metabolism to Triple Negative Breast Cancer Signature
Source: Cancers (Basel). 2021 Nov 23;13(23):5871. doi: 10.3390/cancers13235871 (PMC8657222; doi:10.3390/cancers13235871)
Supplement: Supplementary file 1 [file cancers-13-05871-s001.zip › cancers-1433643-supplementary.pdf]

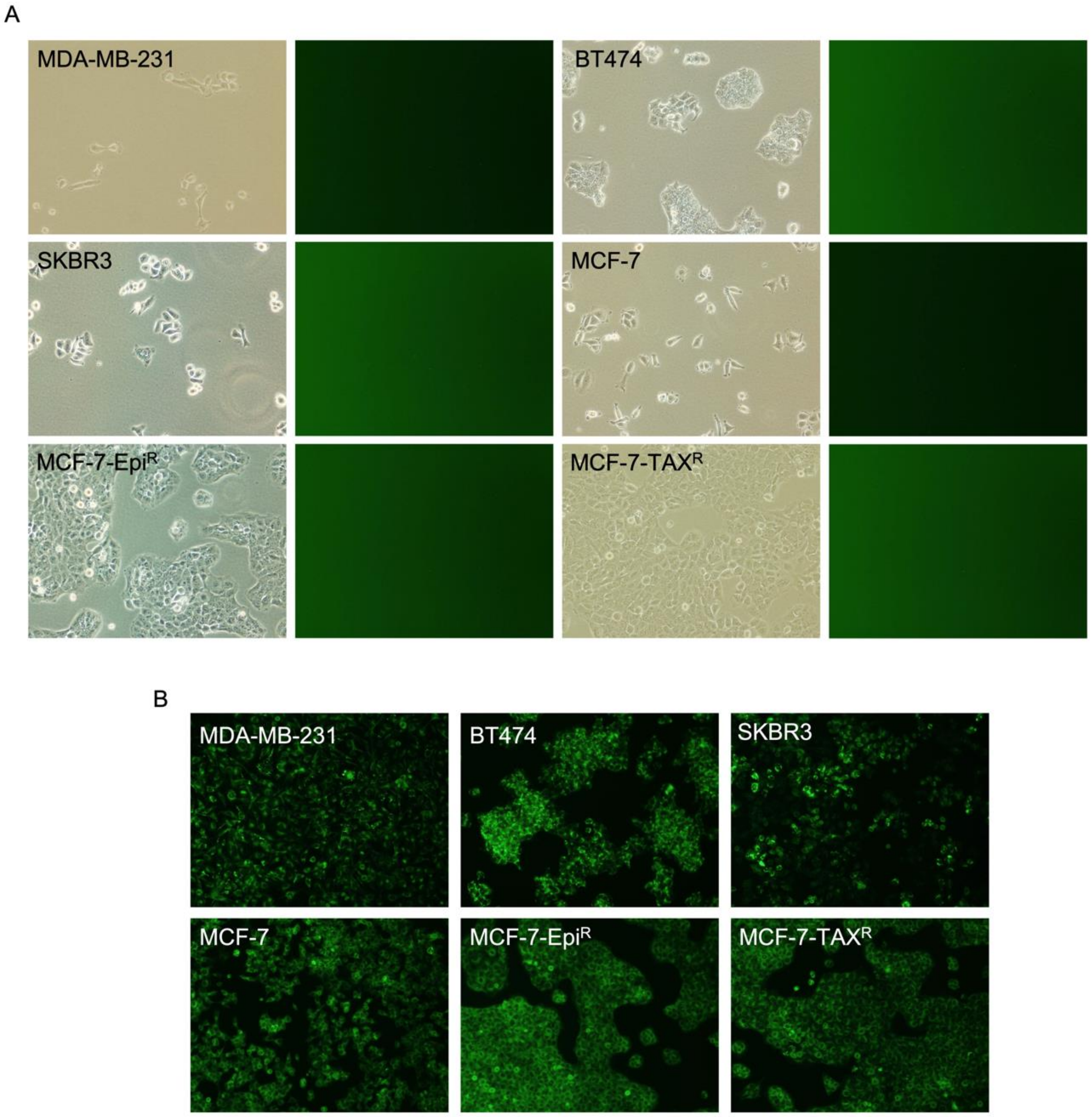

Supplementary Figure S1: Negative and positive control of PA–BODIPY labelling in BCC. A) Negative control: BCC intrinsic PA–BODIPY labelling (background) was measured to corroborate that there was not initial label of this lipid within these cell lines. B) Positive control: a well–known concentration of PA–BODIPY was added in order to ensure that the lipid was internalized into the BCC lines.
